# Reconstructive Surgical Intervention for Malunited Distal End Radius and Ulna Fracture: A Case Report Demonstrating Improved Patient Outcomes

**DOI:** 10.7759/cureus.54328

**Published:** 2024-02-16

**Authors:** Hardik Patel, Aditya Pundkar, Sandeep Shrivastava, Ankit M Jaiswal, Rohan Chandanwale

**Affiliations:** 1 Department of Orthopaedics, Jawaharlal Nehru Medical College, Datta Meghe Institute of Higher Education and Research (Deemed to be University), Wardha, IND

**Keywords:** distal radioulnar joint disruption, bone graft, proximal interphalangeal joint dislocation, malunited distal end ulna fracture, malunited distal end radius fracture

## Abstract

This case report explores the efficacy of reconstructive surgical intervention in addressing malunited fractures of the distal end of the radius and ulna. The study presents a detailed analysis of a specific case, highlighting the surgical techniques employed and their impact on patient outcomes. The report emphasizes the importance of precision in addressing malunited fractures and showcases how the intervention led to improved patient outcomes. By documenting this case, the study contributes valuable insights into the field of orthopedic surgery, providing a basis for further research and enhancing the understanding of optimal approaches to managing such complex fractures.

## Introduction

The incidence of distal radius fractures is 100-190 per 100,000 population per year in males and 282-458 per 100,000 population per year in females. It may most commonly occur in old age due to a trivial fall on an outstretched hand. Conservative management of a distal end radius fracture may result in malunion [1]. Following conservative treatment, morbidity is estimated to be greater than following surgical treatment [2]. Numerous clinical symptoms, such as post-traumatic arthritis, neuropathy, cosmetic deformity, and poor functional results, can be experienced by patients with distal radius malunion. In certain instances, treatment strategies ought to focus more on the symptoms than the findings of imaging tests [3]. Cartilage, bone, or bone substitutes are needed to cushion the bone loss resulting from osteotomy surgery [4]. In order to prevent joint stiffness, post-operative rehabilitation should begin as soon as possible, depending on the fixation [5].

## Case presentation

**Figure 1:**
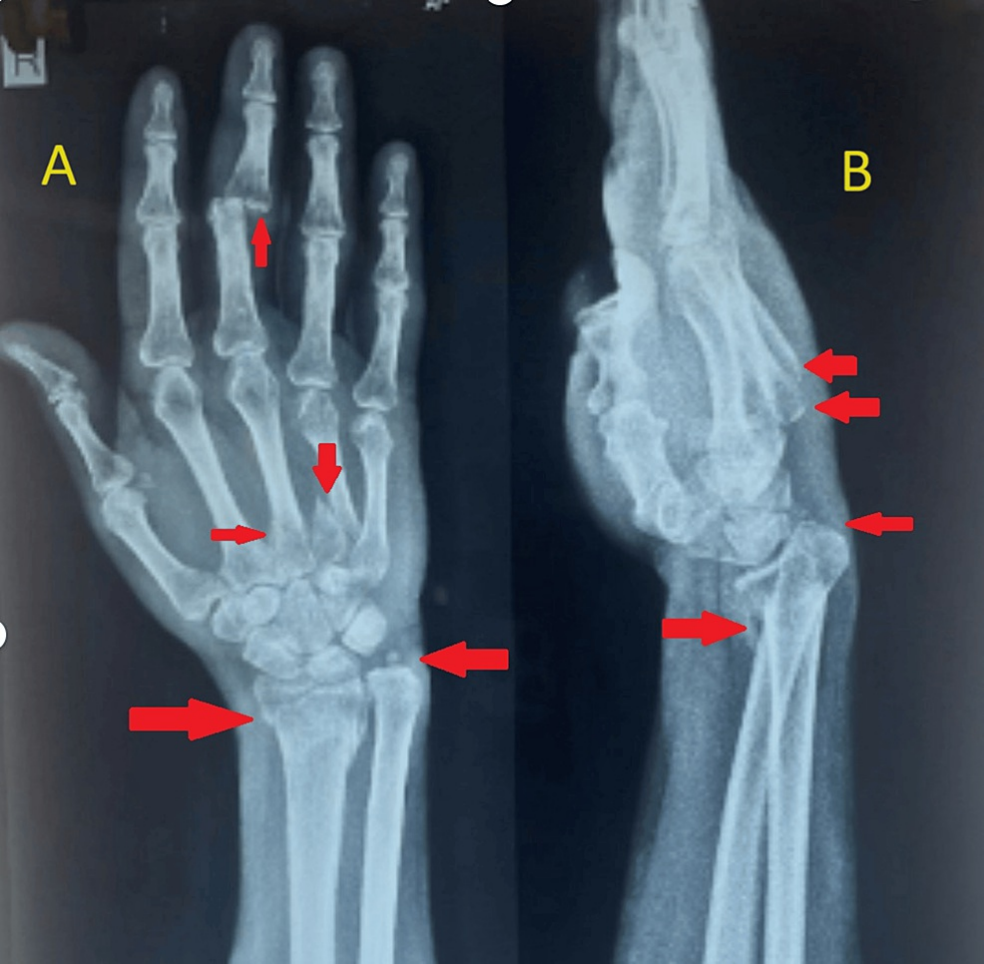
X-ray of the right wrist with hand anteroposterior (A) and lateral (B) views (A) shows the malunited distal end radius fracture with ulna styloid fracture and distal radioulnar joint disruption on the right side, the malunited base of the 4^th^ metacarpal fracture on the right side, the united 3^rd^ metacarpal fracture, and fracture dislocation of the proximal interphalangeal (PIP) joint of the 3^rd^ digit of the right hand. (B) shows the distal radioulnar joint disruption, positive ulnar variance, and malunited 3^rd^ and 4^th^ metacarpal fractures.

A 52-year-old male had suffered from a serious distal radius deformity for two and a half months. The patient allegedly had a history of a road traffic accident due to slipping and falling from a bike, fully extending his hand, approximately two and a half months prior, resulting in injury to the right wrist and hand. After the fall, the patient experienced sudden onset, non-progressive, sharp shooting pain in the right wrist and hand, which persisted continuously throughout the day and was localized to the right wrist and hand. The patient was unable to perform any movements with the right hand. The patient went to a private hospital where an X-ray was performed and surgery was advised, but the patient declined and sought management from a local practitioner, who applied indigenous ointment with a compression bandage. Subsequently, malunion of the right wrist and deformity of the hand developed gradually. The right wrist exhibited severe deformity with a deformity at the 3^rd^ proximal interphalangeal (PIP) joint of the hand. At the time of physical examination, pronation and supination of the right forearm were restricted to 60° and 65°, respectively. Wrist flexion and dorsal extension were limited to 40° and 20°, respectively. Radial and ulnar deviation of the right wrist joint were restricted to 10° each. Grip strength was unable to be assessed due to the deformity at the 3^rd^ PIP joint. The function of the affected extremity was assessed by the Quick DASH (Disability of Arm Shoulder and Hand) score, ranging from 0 (normal function) to 100 (upper limb unusable). The preoperative score of the affected limb was 49. Our patient also underwent standard lateral and posteroanterior X-rays of the right wrist. X-ray of the right wrist with hand anteroposterior and oblique views showed a malunited distal end radius fracture with ulna styloid fracture and disruption of the distal radioulnar joint on the right side, as well as malunited 4^th^ metacarpal fracture and united 3^rd^ metacarpal fracture on the right side. Additionally, a fracture dislocation of the PIP joint of the 3^rd^ digit of the right hand was observed (Figure 1).

**Figure 2:**
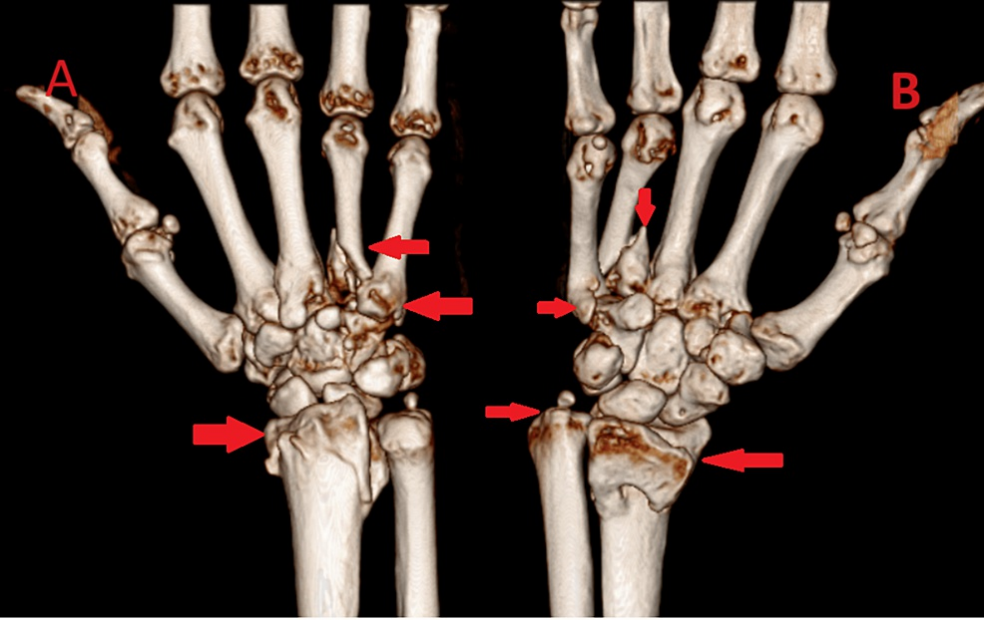
Computed tomography (CT) scan and 3-dimensional reconstruction image of the right wrist with hand ventral (A) and dorsal (B) views (A, B) show the malunited distal radius fracture with ulna styloid fracture, the malunited base of the 4^th^ metacarpal fracture on the right side, and the united 3^rd^ metacarpal fracture on the right side.

**Figure 3:**
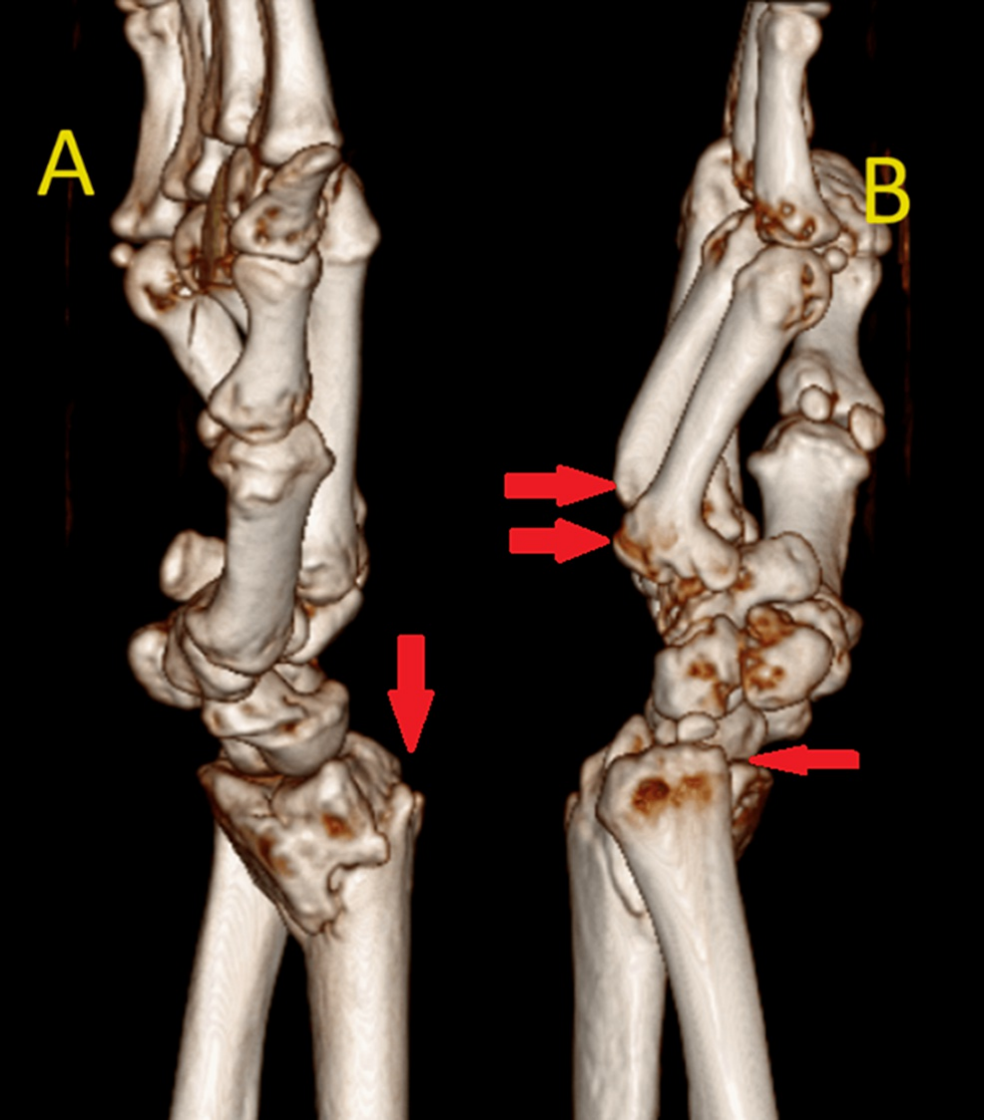
Computed tomography (CT) scan and 3-dimensional reconstruction image of the right wrist with hand lateral (A) and medial (B) views (A, B) show the malunited distal end radius fracture and the malunited base of the 4^th^ metacarpal fracture on the right side.

High-resolution computed tomography (CT) scans and 3-dimensional reconstruction images were used to develop the best surgical plan (Figure 2 and Figure 3).

The patient was planned for open reduction and internal fixation with plate osteosynthesis and bone grafting for the malunited distal end radius fracture, as well as cannulated cancellous (CC) screw fixation for the correction of distal radioulnar joint disruption.

**Figure 4:**
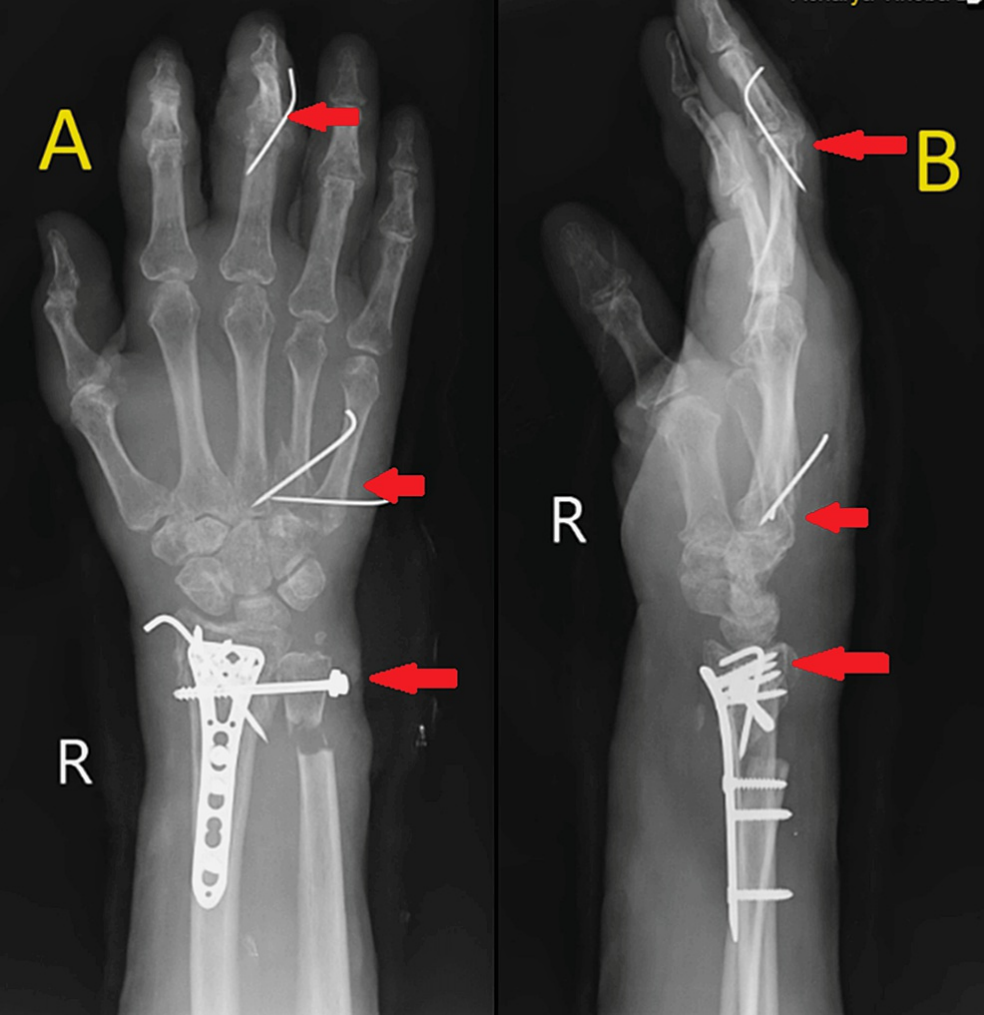
Postoperative X-ray of the right wrist with hand anteroposterior (A) and lateral (B) views (A, B) show open reduction and internal fixation with plate osteosynthesis and bone grafting for the malunited distal end radius fracture and cannulated cancellous (CC) screw fixation for the distal radioulnar joint disruption.

The patient was positioned supine on the operating table. Under nerve block, with tourniquet control and observing all aseptic precautions, the right upper limb was painted and draped. A roughly 5 cm incision was made over the radial aspect of the right forearm, extending 7 cm proximally from the anatomical snuff box. Following this, soft tissue dissection was performed, and the abductor pollicis longus and extensor pollicis brevis were retracted. Upon exploration, a malunited distal end radius was identified. Subsequently, a trans-epiphyseal open wedge osteotomy of the distal end of the radius was performed, and the articular surface of the distal end radius was elevated. A bone graft was harvested from the right iliac crest and used for augmentation. The fracture was then fixed with bone grafting and a volar locking plate, secured by two proximal locking screws, one proximal cortical screw, and five distal cortical screws. A 3 cm direct incision was made over the distal ulna. The skin, superficial fascia, and deep fascia were dissected, revealing positive ulnar variance. Attempts at reduction with traction and manipulation proved unsatisfactory, leading to the extraction of a bone graft from the ulna. Subsequently, the distal radioulnar joint was reduced and temporarily fixed with Kirschner wires (K-wires). Later, it was permanently fixed using one CC screw with a washer. The reduction was confirmed under C-arm guidance and found to be satisfactory. Ulnar cuff resection osteotomy was performed on the distal one-third of the ulna, 2 cm proximal from the ulna styloid process. Thorough irrigation was administered followed by closure in layers. An above-elbow dorsal slab was applied in supination, and the entire procedure was uneventful (Figure 4).

Procedure

Closed reduction and internal fixation with a K-wire fixation were performed for the base of the 4^th^ and 5^th^ metacarpal fracture and dislocation of the PIP joint of the 3^rd^ digit of the right hand.

Under C-arm guidance, a fracture at the base of the 4^th ^and 5^th^ metacarpals was observed, along with the disruption of the PIP joint of the 3^rd^ digit. A 1 cm skin incision was made over the base of the 4^th^ and 5^th^ metacarpals, and with traction and manipulation, reduction was achieved for the fractures, subsequently fixed with K-wires.

**Figure 5:**
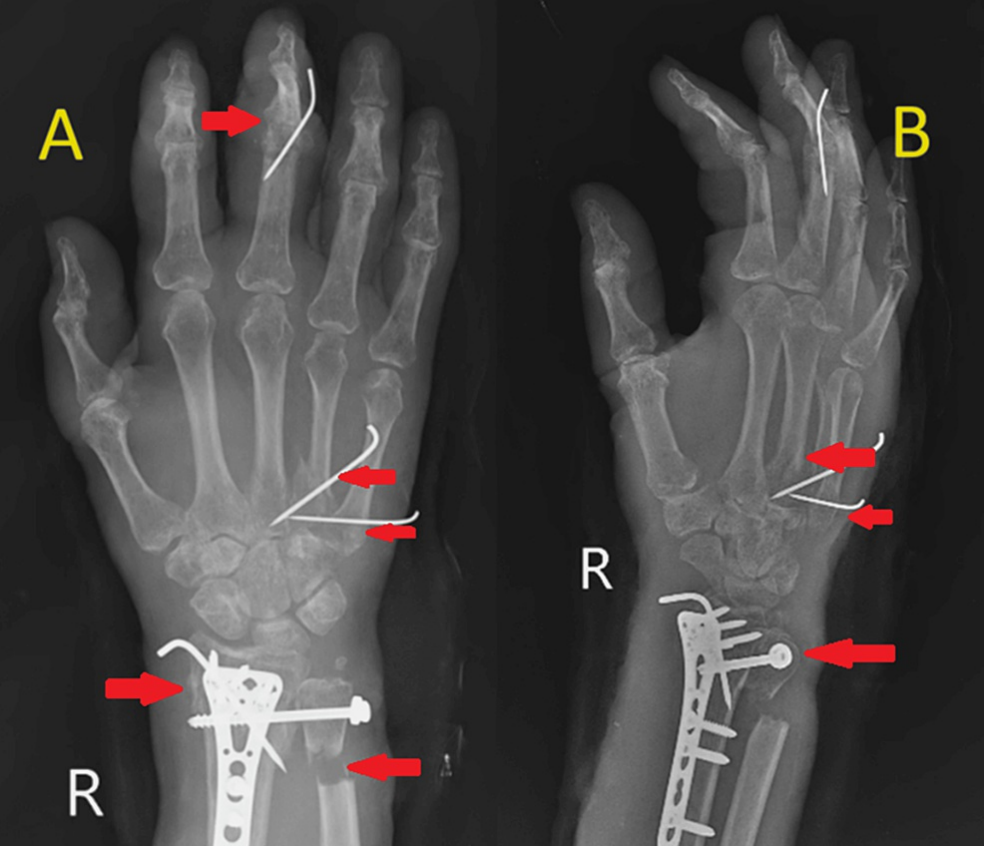
Postoperative X-ray of the right wrist with hand anteroposterior (A) and oblique (B) views (A, B) show closed reduction and internal fixation with a K-wire fixation for the base of the 4^th^ and 5^th^ metacarpal fracture and dislocation of the proximal interphalangeal joint of the 3^rd^ digit of the right hand.

Attempts were made to reduce the dislocation of the PIP joint of the 3^rd^ digit with traction and manipulation but were unsuccessful. A 2 cm skin incision was then made over the lateral side of the PIP joint. After incising the skin, soft tissue dissection was performed, and the transverse retinaculum was incised. The lateral slip of the extensor retinaculum was split to visualize the dislocated PIP joint of the 3^rd^ digit. Soft tissue curettage was conducted at the dislocation site, and with manipulation, reduction was achieved and fixed with a K-wire. A tendon graft was harvested from the palmaris longus, and tendon reconstruction for the lateral collateral ligament was performed using the box loop ligament reconstruction technique. The reduction was confirmed under C-arm guidance and found to be satisfactory. Thorough irrigation was administered followed by closure in layers, and a sterile dressing was applied. The procedure was uneventful, and the patient was shifted to the recovery room for observation. Postoperatively, an above-elbow slab was applied (Figure 5).

On postoperative day 2, sterile dressing was performed, revealing a healthy suture site. The oxygen saturation (SpO_2_) in all fingers ranged between 97-99%, and finger movements were present, indicating adequate distal circulation. A postoperative X-ray was conducted and found to be satisfactory. Physiotherapy was initiated in the form of shoulder range of motion (ROM) exercises and active finger movement exercises. On postoperative day 7, another sterile dressing was done, and the suture site was observed to be in a healthy condition. By postoperative day 12, suture removal was carried out and found to be a healthy process. The patient remained vitally stable throughout the observation period. Upon discharge, advice was given to continue the above-elbow slab application for the next three weeks. The patient was instructed to return to the outpatient department after this period for the removal of K-wires and further mobilization of the hand and wrist.

Surgical intervention can significantly improve patient outcomes compared to conservative management. By addressing the malunion through reconstructive surgery, we were able to restore proper alignment and function of the wrist and hand. Additionally, early mobilization post-surgery was emphasized to reduce the risk of joint stiffness and promote better recovery. By encouraging early movement, we aimed to facilitate the restoration of wrist and finger ROM, which is essential for the patient's overall functionality. Through comprehensive surgical management and early mobilization, we were able to restore normal anatomy and function, highlighting the importance of timely and appropriate medical care for complex fractures.

## Discussion

Malunion as a complication is commonly seen in cases of distal end radius fractures that were treated by conservative management, which is more commonly observed in unstable distal end radius fractures [6].

Normal alignment and congruity at the articular surface are important to achieve a good functional outcome [7]. A study by Kapandji et al. presents a technique of internal fixation by pinning for extra-articular distal end radius fractures; however, in unstable or comminuted fractures, these techniques can be followed by secondary displacement with excessive rotation, ultimately resulting in malunion [1,8]. Postoperatively, mainly due to the poor quality of anatomical reduction, ulnar translation of the radial epiphysis resulted in the loss of pronation. Radial shortening decreased both pronation and supination [9]. Dorsal and volar displacement fractures further present with angulated malunion if not managed properly. However, limited features of anterior malunion, in which there is a limitation of volar angulation due to the anterior capsule plane with minimal shortening of the radius and pronated distal fragment, are responsible for distal radioulnar joint disruption with a gross decrease in supination. Various treatment modalities have been reviewed, with the main goal of achieving normal anatomy [6].

There are various surgical options available nowadays, with the most reported being radial corrective osteotomy with ulnar shortening osteotomy [3,10,11]. There are instances of carpal fractures occurring concurrently with distal radius fractures [10]. Corrective osteotomy with volar and dorsal fixation could correct volar tilt deformities and increase wrist joint stability. This can effectively relieve wrist pain, improve function, and increase grip strength, resulting in positive long-term functional outcomes [11].

With patient-specific guides and printed titanium plates that are customized for each patient, planned 3-dimensional corrective osteotomies can be used to treat distal radius and forearm malunions [12]. Their patients' function and discomfort significantly improved a year after surgery. They also saw only one small wound-related complication, and the bone alignment could be adjusted to match the unaffected side [12]. Ulnar shortening osteotomy remains the primary treatment option in younger and highly demanding people [13].

## Conclusions

Malunited distal end radius fractures pose significant challenges in treatment and reconstruction. The primary goal is to restore normal anatomy, thereby increasing wrist and finger ROM, while also aiming to reduce pain during movement. In our study, radius osteotomy was combined with ulnar surgery to restore the anatomy of the distal radioulnar joint, a crucial step in optimizing postoperative functional outcomes. Early mobilization, depending on the fixation method used, may help reduce the risk of joint stiffness. Additionally, metacarpal fixation was performed to restore hand function. Surgical management should be the preferred approach in cases of intra-articular, unstable, or comminuted fractures. Moreover, there must be increased awareness in developing countries to discourage the use of indigenous treatment methods, as they may lead to further serious complications.
